# Lnc‐NTF3‐5 promotes osteogenic differentiation of maxillary sinus membrane stem cells via sponging miR‐93‐3p

**DOI:** 10.1111/cid.12553

**Published:** 2017-11-06

**Authors:** Wei Peng, Shuang‐Xi Zhu, Jin Wang, Li‐Li Chen, Jun‐Quan Weng, Song‐Ling Chen

**Affiliations:** ^1^ Department of Oral and Maxillofacial Surgery The First Affiliated Hospital, Sun Yat‐sen University Guangzhou China; ^2^ Guangdong Key Laboratory of Stomatology Sun Yat‐sen University Guangzhou China; ^3^ Department of Pathology The First Affiliated Hospital, Sun Yat‐sen University Guangzhou China; ^4^ Department of Stomatology Shenzhen People's Hospital, Second Clinical Medical School, Jinan University Shenzhen China

**Keywords:** long non‐coding RNAs, microRNAs, maxillary sinus membrane stem cells, osteogenic differentiation

## Abstract

**Background:**

The function and the mechanism of long non‐coding RNAs (lncRNAs) on the osteogenic differentiation of maxillary sinus membrane stem cells (MSMSCs) remain largely unknown.

**Materials and Methods:**

The expression of lnc‐NTF3‐5 and Runt‐related transcription factor 2 (RUNX2), Osterix (OSX), and Alkaline Phosphatase (ALP) was examined by quantitative real‐time PCR (qRT‐PCR) in MSMSCs during the process osteogenic differentiation. Then the function of lnc‐NTF3‐5 was evaluated by loss‐ and gain‐of‐function techniques, as well as qRT‐PCR, western blot, and Alizarin Red staining. In addition, the microRNAs (miRNAs) sponge potential of lnc‐NTF3‐5 was assessed through RNA immunoprecipitation, dual luciferase reporter assay, and in vivo ectopic bone formation.

**Results:**

Lnc‐NTF3‐5, RUNX2, OSX, and ALP increased alone with the differentiation. Inhibition of lnc‐NTF3‐5 decreased the expression of RUNX2, OSX, and ALP both at mRNA and protein levels. Alizarin red staining showed similar trend. In contrast, overexpression of lnc‐NTF3‐5 presented totally opposite effects. Besides, overexpression of lnc‐NTF3‐5 could decrease the expression of microRNA‐93‐3p (miR‐93‐3p). Enhance miR‐93‐3p could also inhibit the expression level of lnc‐NTF3‐5. RNA immunoprecipitation demonstrated that lnc‐NTF3‐5 is directly bound to miR‐93‐3p and dual luciferase reporter assay proved that miR‐93‐3p targets 3′ UTR of RUNX2 to regulate its expression. Ultimately, in vivo bone formation study showed that lnc‐NTF3‐5 and miR‐93‐3p inhibitor co‐transfection group displayed the strongest bone formation.

**Conclusions:**

The novel pathway lnc‐NTF3‐5/miR‐93‐3p/RUNX2 could regulate osteogenic differentiation of MSMSCs and might serve as a therapeutic target for bone regeneration in the posterior maxilla.

## 1 INTRODUCTION

There is a tremendous clinical need for reconstruction of lost craniofacial bone caused by tumor and trauma. Numerous types of mesenchymal stem cells (MSCs) have been found in craniofacial tissues, which have been shown to possess stem cell properties such as self‐renewal, colony formation, and multilineage differentiation capacity.^1^ These cells include stem cells from human exfoliated deciduous teeth (SHED), periodontal ligament stem cells (PDLSCs), dental follicle cells (DFCs), stem cells from apical papilla (SCAP), gingival MSCs (GMSCs), alveolar bone‐derived MSCs (ABMSCs), and oral mucosa stem cells (OMSCs).^1^, ^2^, ^3^ Maxillary sinus floor elevation is a common procedure to resolve the inadequate bone height in the posterior maxilla.^4^ Clinical studies have shown that maxillary sinus augmentation can be reached by elevating the MSM with or without grafting,^5^ which suggested that maxillary sinus membrane possesses osteogenic activity. Further studies have isolated and characterized maxillary sinus membrane stem cells (MSMSCs) in maxillary sinus membrane, which displayed stem cell properties similar to bone marrow mesenchymal stem cells (BMMSCs).^6^ Our previous study demonstrated that MSMSCs could express MSC markers such as STRO‐1, CD146, CD29. and CD44.^6^ These cells can form colonies and differentiate into adipocytes, osteoblasts, and chondrocytes.^6^. Evidence further showed that MSMSCs can differentiate into osteoblasts both in vitro and in vivo,^7^, ^8^, ^9^, ^10^ which represented a useful cell source of MSCs for bone tissue engineering.

Despite several researches on MSMSCs differentiation, the precise molecular mechanisms of osteogenic differentiation remain unclear. Osteogenic differentiation of MSCs is controlled by a variety of signaling pathways.^11^ Recently, long non‐coding RNAs (lncRNAs) have emerged as important players in controlling stem cells fate and behavior.^12^ For instance, a preliminary research found that a number of lncRNAs were significantly altered during the osteogenic differentiation of C3H10T1/2 cells.^13^ Anti‐differentiation ncRNA (ANCR) functions as a suppressor of osteogenic differentiation through a pathway suggested to involve enhancer of zeste homolog 2 (EZH2) and RUNX2.^14^ Huang et al. revealed a H19/miR‐675/TGF‐β1/Smad3/HDAC pathway in regulating osteogenic differentiation of human MSCs.^15^ In addition, lncRNA‐POIR^16^ and MEG3^17^ were reported to regulate osteogenic differentiation through different mechanisms, whereas knockdown of HoxA‐AS3 expression enhanced osteogenic differentiation and osteogenesis markers expression in MSCs.^18^ These studies implied that lncRNAs both positively and negatively control the differentiation state of stem cells. Thus, the role of lncRNAs in MSMSCs differentiation is a great worthy studying question.

In our previous research, differential lncRNAs expression profiles in MSMSCs during osteogenic differentiation were established via microarray, and several upregulated lncRNAs between undifferentiated and osteogenic differentiated MSMSCs have been identified.^19^ LncRNA‐MODR/miR‐454/RUNX2 pathway was suggested to regulate osteogenic differentiation of MSMSCs.^19^ Meanwhile, lnc‐NTF3–5 (ENST0000537192.1) displayed a significantly increase after 7 d of induction. Nevertheless, the function and the underlying molecular mechanism of lnc‐NTF3–5 regulates the osteogenic differentiation of MSMSCs are still unknown.

To address these questions, this study aims to explore the role of lnc‐NTF3–5 in osteogenic differentiation of MSMSCs and how it regulates the process. This study may provide a new molecular mechanism for osteogenic differentiation of MSMSCs and a new therapeutic target for bone regeneration in the posterior maxilla.

## 2 MATERIALS AND METHODS

### 2.1 Informed consent

Studies were conducted in accordance with the guidelines of the Medical Ethics Committee of Sun Yat‐Sen University and approved by the Sun Yat‐Sen University Joint Institutional Review Board (IACUC‐DB‐16–1212). All donors provided informed written consent prior to specimen collection.

### 2.2 Cell culture

HEK‐293T cells were purchased from the American Type Culture Collection (ATCC). MSMSCs were isolated from normal human MSM according to our previously published methods.^6^ Briefly, tissues were minced and digested with 3 mg/mL collagenase type I (Sigma, St. Louis, Missouri) and 4 mg/mL dispase (Roche, Mannheim, Germany) for 1 h at 37°C. Then the samples were passed through a 70 μm strainer (Falcon, BD Labware, Franklin Lakes, New Jersey). Cells were supplemented into 75‐cm^2^ culture flasks (Costar, Cambridge, Massachusetts) with alpha modification of Eagle's medium (GIBCO BRL, Grand Island, New York), and then incubated in 5% CO_2_ and 37°C.

### 2.3 Osteogenic differentiation

Osteogenic differentiation was conducted according to the instructions from the manufacturer (Invitrogen, Carlsbad, California). MSMSCs were plated into 6‐well plates (Corning Life Sciences, Tewksbury, Massachusetts) at a density of 1 × 10^4^ cells/well. BMP2 (100 ng/mL) was used to induce the differentiation when cells were reached 80% confluence.

### 2.4 Quantitative real‐time PCR

RNA was extracted from cells via TRIzol reagent (Invitrogen, Carlsbad, California), then cDNA was synthesized with SuperScript III reverse transcriptase kit (Qiagen, Valencia, California) according to the instruction from the manufacturer. The qRT‐PCR was performed with TaqMan Universal PCR master mix (Applied Biosystems, Foster City, California) as described previously.^6^ Relative mRNA and lncRNA expression were calculated with normalization to GAPDH, and U6 was used to normalize expression of miRNA. The primers used for amplification are listed in Table 1.

**Table 1:** The specific primer sequences for qRT‐PCR

ID	Sequence (5′–3′)
lnc‐NTF3–5 F	ATCAGCGAGACTCCGTGG
lnc‐NTF3–5 R	GGAAATGGTGGAAATGTGC
RUNX2 F	TACTATGGCACTTCGTCAGGA
RUNX2 R	GATTCATCCATTCTGCCACTA
OSX F	CCTCTGCGGGACTCAACAAC
OSX R	AGCCCATTAGTGCTTGTAAAGG
ALP F	CCAGGACATCGCTACGCA
ALP R	CCACCTTGGCTGTAGTCATCTG
miR‐93‐3p F	ACACTCCAGCTGGGACTG
miR‐93‐3p R	CTCAACTGGTGTCGTGGA
GAPDH F	TGTTCGTCATGGGTGTGAAC
GAPDH R	ATGGCATGGACTGTGGTCAT
U6 F	CTCGCTTCGGCAGCACA
U6 R	AACGCTTCACGAATTTGCGT

### 2.5 Western blot

Cells were washed with precooled PBS and lysed using RIPA buffer (Beyotime, Beijing, China). Then proteins were separated by 12% sodium dodecyl sulfate polyacrylamide gel electrophoresis (SDS‐PAGE) and transferred to polyvinylidene difluoride (PVDF) membrane. Appropriate primary antibodies against RUNX2 (Abcam, Cambridge, UK), OSX (Santa Cruz Biotechnology, Santa Cruz, California), ALP (Santa Cruz Biotechnology, Santa Cruz, California), and GAPDH (Cell Signaling Technology, Danvers, Massachusetts) incubated respectively overnight at 4°C. Then membranes were incubated with relevant secondary antibodies and visualized using ECL Western blotting detection kit (Thermo Fisher Scientific, Rockford, Illinois). Results of western blotting were analyzed with Gel Image system software (Tanon, Shanghai, China).

### 2.6 Alizarin red staining

Cells were seeded into 6‐well plates at a density of 2 × 10^4^ cells/well contain control or osteogenic media. After the indicated number of days of culture, cells were stained with Alizarin Red S (Sigma, St. Louis, Missouri) according to the instruction from the manufacturer and imaged using an inverted Zeiss AxioObserver Z1 microscope (Carl Zeiss Meditec, Inc, Dublin, California). Alizarin Red staining quantification was performed according to our previously published methods.^9^ One milliliter of 10% cetylpyridinium chloride (Sigma, St. Louis, Missouri) was added to each well for 45 min. The absorbance was read at 562 nm.

### 2.7 Plasmids, lentiviruses, and transfection

For knockdown of lnc‐NTF3–5, the shRNA sequence targeting lnc‐NTF3–5 (sh‐lnc‐NTF3–5) and a scrambled shRNA sequence (scrambled) was cloned into pENTR/U6 vector (Invitrogen, Carlsbad, California). The target sequence for sh‐lnc‐NTF3–5 is 5′‐TAGGCCAACCAACACAATTCATA‐3′. All constructs were verified by sequencing. The MSMSCs were transfected with Lipofectamine™ 2000 (Invitrogen, Carlsbad, California) following the protocol from the manufacturer.

For overexpression of lnc‐NTF3–5, the full length of lnc‐NTF3–5 was cloned into pLKD‐CMV‐G lentiviral vector (Obio Technology Corp, Ltd, Shanghai, China). Then the lnc‐NTF3–5 plasmid vector (lnc‐NTF3–5) or empty plasmid vector (vector) and the packaging plasmids Helper 1.0 and Helper 2.0 (Obio Technology Corp, Ltd, Shanghai, China) were transfected into MSMSCs. Lentiviral infection was conducted as described previously.^19^

For miRNA function study, mimics for miR‐93‐3p, miR‐93‐3p inhibitor, as well as a nontargeting control miRNA mimic (nc RNA), were obtained from RiboBio (Guangzhou, Guangdong, China). Then MSMSCs were transfected in the presence of 8 mg/mL Polybrene (Santa Cruz Biotechnology, Dallas, Texas) overnight.

### 2.8 Luciferase assays

The 1 × 10^4^ 293T cells in 24‐well plates were plated. A wild‐type lnc‐NTF3–5 (lnc‐NTF3–5 WT, with putative miR‐93‐3p target binding sequence) or mutated (lnc‐NTF3–5 Mut, with mutation of individual bases in the binding site) plasmid was constructed with pmirGLO luciferase vectors (Promega, Madison, Wisconsin). A wild‐type or mutated RUNX2 3′ UTR reporter plasmid were constructed with psiCHECK‐2‐derived reporter vectors (Promega, Madison, Wisconsin). 293T cells were transfected with 1 µg of the reporter constructs and 200 ng of Renilla luciferase internal control vector using Lipofectamine 2000 (Invitrogen, Carlsbad, California). The luciferase activity was measured 48 h after transfection using the Dual Luciferase Reporter Assay System (Promega, Madison, Wisconsin) according to the instructions from the manufacturer. Each experiment was repeated at least three times.

### 2.9 RNA immunoprecipitation (RIP)

RIP assay was performed with Magna RIP RNA‐Binding Protein Immunoprecipitation Kit (Millipore, Billerica, Massachusetts) in accordance with the instructions from the manufacturer. Briefly, magnetic beads were incubated with antibody against Ago2 (Millipore, Billerica, Massachusetts) and mouse IgG control. RNA was extracted and relative expression levels of lnc‐NTF3–5 and miR‐93‐3p in extract were detected by qRT‐PCR as described above.

### 2.10 RNA fluorescence in situ hybridization (RNA‐FISH)

Lnc‐NTF3–5, 18s, U6 subcellular localization in MSMSCs was conducted with a FISH kit (RiboBio, Guangzhou, Guangdong, China). Briefly, MSMSCs were fixed with 4% paraformaldehyde before prehybridized with hybridization solution and then incubated with a digoxigenin‐labeled lnc‐NTF3–5, 18s, U6 probe. The antisense probe served as a negative control. Images were acquired via an LSM 700 Confocal Laser Scanning Microscope (Carl Zeiss, Thornwood, New York).

### 2.11 In vivo transplantation

Animal experiments in this study were conducted in accordance with protocols approved by the Ethical Committee on Animal Research of the Sun Yat‐Sen University. BALB/c‐nu male mice (5‐wk‐old, *n* = 8 for each group) were purchased from the Experimental Animal Department of the Chinese Academy of Sciences, and were housed in SPF conditions during the whole experiment. MSMSCs were transfected with (1) control group, (2) lnc‐NTF3–5, (3) lnc‐NTF3–5 and miR‐93‐3p mimic, and (4) lnc‐NTF3–5 and miR‐93‐3p inhibitor 2 d before transplantation. Approximately 4 × 10^6^ MSMSCs were combined with 40 mg of deproteinized bovine bone (Bio‐Oss; Geistlich, Wolhusen, Switzerland) and then placed in the subcutaneous pockets as described in previous studies.^20^ Briefly, after the mice were anesthetized, approximately 2 cm incisions were made on the back of mice. Then the four groups of constructs were transplanted subcutaneously. The mice were sacrificed, and the transplants were harvested after 8 wk for further studies as described in our studies.^9^ The transplants were fixed with 10% buffered formalin and decalcified with 10% ethylenediaminetetraacetic acid (pH 8.0) for 2 wk. Then the grafts were embedded in paraffin, sectioned into 3 μm sections, and stained with hematoxylin and eosin (H&E). The percentage of new bone formation per total section area was calculated using the average value of the three randomly selected parallel slices with Image Pro Plus 5.0 system (Media Cybernetics, Silver Springs, Maryland).

### 2.12 Statistical analyses

All results were presented as the mean ± standard deviations (SD) from at least in triplicate and were analyzed using two‐tailed unpaired Student's *t*‐test. The *P* values were adjusted using the Bonferroni correction for post hoc analysis for multiple group analysis. A *P* value < .05 was considered statistically significant.

## 3 RESULTS

### 3.1 Lnc‐NTF3–5 expression was correlated with osteogenic differentiation of MSMSCs

**Figure 1:**
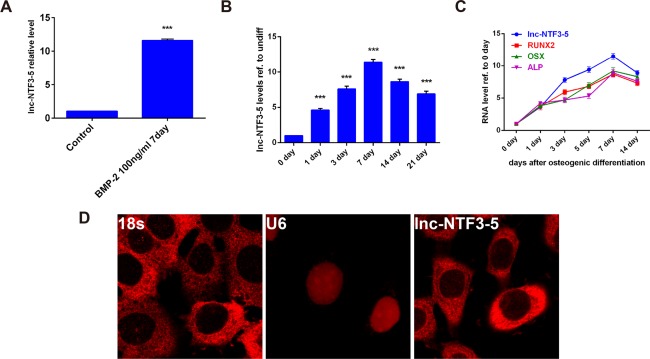
Lnc‐NTF3–5 expression during osteogenic differentiation of MSMSCs. (A) Relative expression of lnc‐NTF3–5 after 7 d of treatment by BMP‐2. (B) Time‐dependent expression of lnc‐NTF3–5 as determined by qRT‐PCR analysis during osteogenic differentiation of MSMSCs. (C) Correlation analysis between lnc‐NTF3–5 levels and RUNX2, OSX, and ALP expression levels in MSMSCs. (D) RNA‐FISH was conducted to detect lnc‐NTF3–5 localization. 18S and U6 probe were also hybridized to confirm RNA‐FISH specificity. The data are presented as the mean ± SD (*P < .05, **P < .01, ***P < .001)

In our previous microarray result, ENST0000537192.1 (lnc‐NTF3–5) was significantly raised after inducing with 100ng/ml of BMP‐2 for 7 d in MSMSCs. To confirm the expression level of lnc‐NTF3–5 in osteogenic differentiated MSMSCs, qRT‐PCR was conducted and results showed that BMP‐2 induced a 10‐fold increase on day 7 compared with the uninduced control (Figure 1A). To evaluate the correlation of lnc‐NTF3–5 with osteogenic differentiation, temporal lnc‐NTF3–5 expression was analyzed. With induction, lnc‐NTF3–5 expression level increased significantly from day 0 to day 7 and reached the maximal expression on day 7 (Figure 1B). Then a declining trend from day 14 to day 21 was observed (Figure 1B). Consistent with lnc‐NTF3–5, osteogenic differentiation marker genes such as Runt‐Related Transcription Factor (RUNX2), Osterix (OSX), and Alkaline Phosphatase (ALP) were also upregulated from day 0 to day 7 and declined on day 14 (Figure 1C). As indicated by RNA‐FISH assay, lnc‐NTF3–5 was found to be distributed in cytoplasm of MSMSCs, which was similar to 18S expression. In contrast, U6 was located in the nucleus (Figure 1D). RNA‐FISH results suggested that lnc‐NTF3–5 exert its function in cytoplasm.

### 3.2 Lnc‐NTF3–5 upregulation promoted osteogenic differentiation of MSMSCs

**Figure 2:**
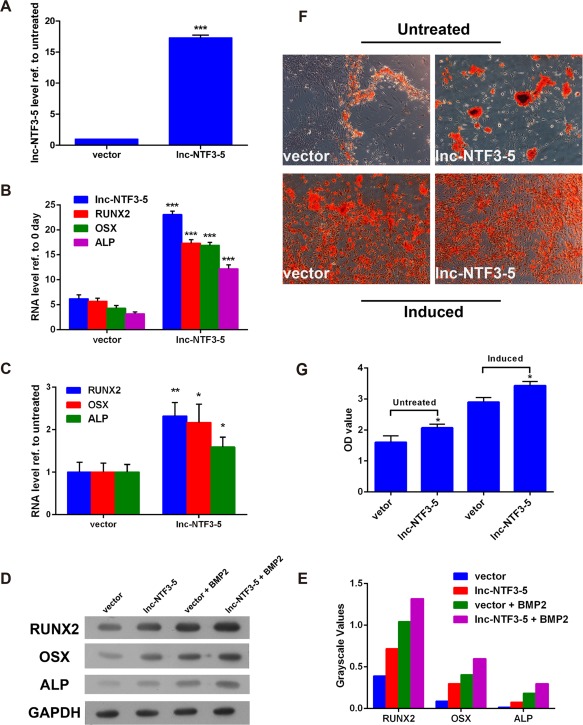
The function of upregulated lnc‐NTF3–5 in osteogenic differentiation of MSMSCs. (A) Relative expression of lnc‐NTF3–5 after transfection of lnc‐NTF3–5 overexpression‐vector in MSMSCs. (B and C) Relative expression of osteogenic markers in mRNA level after transfected by lnc‐NTF3–5‐overexpression‐vector in undifferentiated and osteogenic differentiated MSMSCs, respectively. (D) Western blot analysis of osteogenic markers in undifferentiated and osteogenic differentiated MSMSCs after transfection of lnc‐NTF3–5 overexpression‐vector. (E) Results of western blotting were analyzed with Gel Image system software (Tanon, Shanghai, China) and data were presented as ratio of target protein to GAPDH in the form of grayscale value. (F) Alizarin red S staining of osteogenic differentiated MSMSCs after transfection of lnc‐NTF3–5 overexpression vector. (G) Quantitative comparison of mineralized nodules in different groups using cetylpyridinium chloride. OD, optical density (*P < .05, **P < .01, ***P < .001)

Combined with previous observations, we speculated that lnc‐NTF3–5 can stimulate osteogenic differentiation of MSMSCs induced with BMP‐2. To prove this hypothesis, lentiviral vector was constructed to overexpress lnc‐NTF3–5 in MSMSCs. The results justified our conjecture. The expression level of lnc‐NTF3–5 was increased to more than 15‐fold higher than the vector group (Figure 2A). After cultured in undifferentiation medium for 7 d, qRT‐PCR analysis revealed that lnc‐NTF3–5‐overexpression significantly increased RUNX2, OSX, and ALP mRNA levels and protein levels (Figure 2B, D, E). Meanwhile, the expression of RUNX2, OSX, and ALP was upregulated to a great extent in MSMSCs induced with osteogenic differentiation medium (Figure 2C–E). In addition, the overexpression of lnc‐NTF3–5 led to an enhancement of mineralized bone matrix assessed by alizarin red staining (Figure 2F, G).

### 3.3 Lnc‐NTF3–5 knockdown decreased osteogenic differentiation of MSMSCs

**Figure 3:**
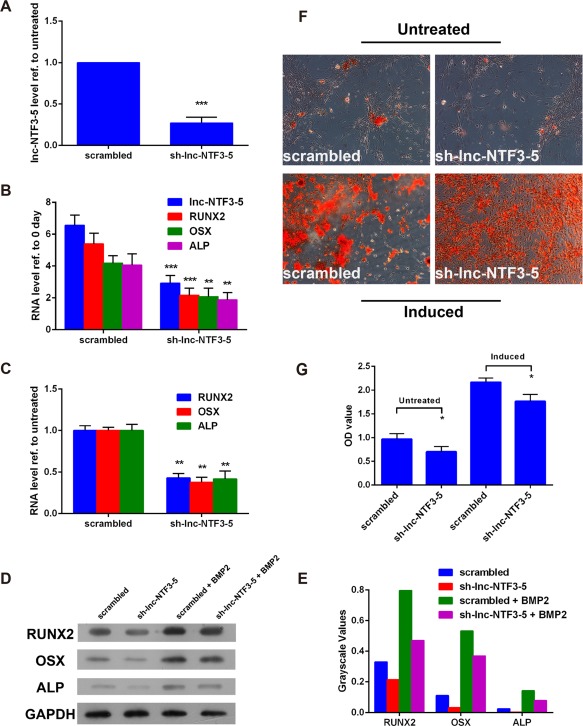
The function of downregulated lnc‐NTF3–5 in osteogenic differentiation of MSMSCs. (A) Relative expression of lnc‐NTF3–5 after transfection of sh‐lnc‐NTF3–5 vector in MSMSCs. (B and C) Relative expression of osteogenic markers in mRNA level after transfection of sh‐lnc‐NTF3–5 vector in undifferentiated and osteogenic differentiated MSMSCs, respectively. (D) Western blot analysis of osteogenic markers after transfection of sh‐lnc‐NTF3–5 vector in undifferentiated and osteogenic differentiated MSMSCs. (E) Results of western blotting was analyzed with Gel Image system software (Tanon, Shanghai, China) and data were presented as a ratio of target protein to GAPDH in the form of grayscale value. (F) Alizarin red S staining of osteogenic differentiated MSMSCs after transfection of sh‐lnc‐NTF3–5 vector. (F) Quantitative comparison of mineralized nodules in different groups using cetylpyridinium chloride. OD, optical density (*P < .05, **P < .01, ***P < .001)

Furthermore, we also investigated the effect of lnc‐NTF3–5 downregulated on osteogenic differentiation of MSMSCs. We constructed a shRNA‐based system to inhibit the expression of lnc‐NTF3–5. The efficiency of the lnc‐NTF3–5 knockdown was confirmed by qRT‐PCR, result showing that the expression of lnc‐NTF3–5 was down by half and lower than the scrambled group (Figure 3A). Contrary to lnc‐NTF3–5 upregulated, lnc‐NTF3–5 knockdown significantly decreased the osteogenic marker RUNX2, OSX, and ALP expression in MSMSCs, either undifferentiation or osteogenic differentiation induced, compared with the scrambled group on day 7 (Figure 3B–E). And, it turned out that sh‐lnc‐NTF3–5 decreased calcium deposition as assessed by alizarin red staining at day 21 (Figure 3F, G). These data implied that lnc‐NTF3–5 can regulate MSMSCs differentiate into osteoblasts.

### 3.4 MiR‐93‐3p is the target of lnc‐NTF3–5

**Figure 4:**
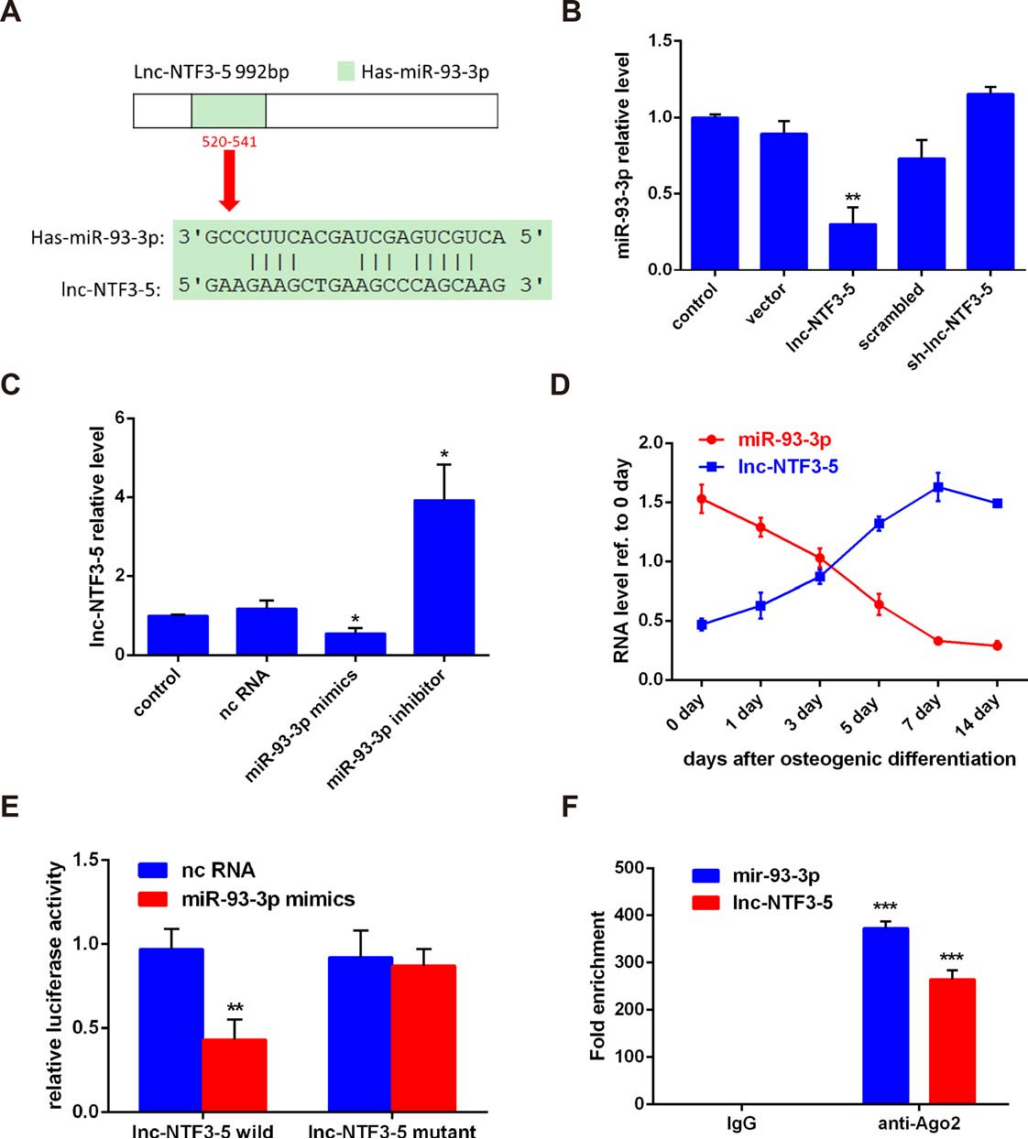
Lnc‐NTF3–5 functions as an endogenous sponge of miR‐93‐3p. (A) The potential binding sites between lnc‐NTF3–5 and miR‐93‐3p predicted by biological software. (B) Relative miR‐93‐3p expression level in MSMSCs transfected with lnc‐NTF3–5 or sh‐lnc‐NTF3–5. (C) Relative lnc‐NTF3–5 expression in MSMSCs transfected with miR‐93‐3p mimics or miR‐93‐3p inhibitor. (D) Correlation analysis between lnc‐NTF3–5 levels and miR‐93‐3p levels in MSMSCs 0, 1, 3, 5, 7, and 14 d after osteogenic differentiation. (E) The target validation using luciferase reporters in 293T cells. The relative luciferase activities of luciferase reporters containing WT or Mut lnc‐NTF3–5 were assayed 48 h after cotransfection with miR‐93‐3p mimics or nc RNA. Relative Renilla luciferase activity was normalized to that of firefly luciferase. (F) Relative expression levels of lnc‐NTF3–5 and miR‐93‐3p in MSMSCs were accessed by RIP assays and qRT‐PCR (*P < .05, **P < .01, ***P < .001)

To further explore the underlying mechanism by which lnc‐NTF3–5 regulates osteogenic differentiation, predicted targets of lnc‐NTF3–5 were analyzed using miRnada, miRDB, and Target Scan software (Tanon, Shanghai, China). The miRNAs identified as targets by three of these software were selected. Among the genes, *miR‐93‐3p* has the highest predictive score and was subjected to further analysis. Results showed that *miR‐93‐3p* might be the potential target of lnc‐NTF3–5 (Figure 4A). Then we evaluated miR‐93‐3p expression in lnc‐NTF3–5‐overexpression and ‐knockdown MSMSCs. Results showed that overexpression of lnc‐NTF3–5 significantly inhibited miR‐93‐3p expression, while knockdown of lnc‐NTF3–5 promoted miR‐93‐3p expression (Figure 4B).

In addition, the expression of lnc‐NTF3–5 in response to the miR‐93‐3p mimics and miR‐93‐3p inhibitor was also examined. QRT‐PCR data revealed that lnc‐NTF3–5 expression was significantly suppressed by miR‐93‐3p mimics (Figure 4C). During the process of osteogenic differentiation from day 0 to day 14, the expression of miR‐93‐3p was negatively correlated with lnc‐NTF3–5 (Figure 4D).

To determine whether lnc‐NTF3–5 directly regulates miR‐93‐3p, we generated luciferase reporter constructs. The miR‐93‐3p mimics and lnc‐NTF3–5 wild‐type or mutant reporter were transferred into 293T cells together. The results showed that the luciferase activity of lnc‐NTF3–5 wild‐type reporter was strongly suppressed by miR‐93‐3p mimics (Figure 4E). However, the lnc‐NTF3–5 mutant reporter was not affected by miR‐93‐3p mimics (Figure 4E). As a final note, lnc‐NTF3–5 and miR‐93‐3p expression levels were significantly higher in the anti‐Ago2 group compared with the anti‐normal IgG group (Figure 4F). RIP results suggested that lnc‐NTF3–5 is associated with the RNA‐induced silencing complex (RISC). These results indicated that lnc‐NTF3–5 and miR‐93‐3p directly regulate each other.

### 3.5 MiR‐93‐3p suppressed the expression of RUNX2

**Figure 5:**
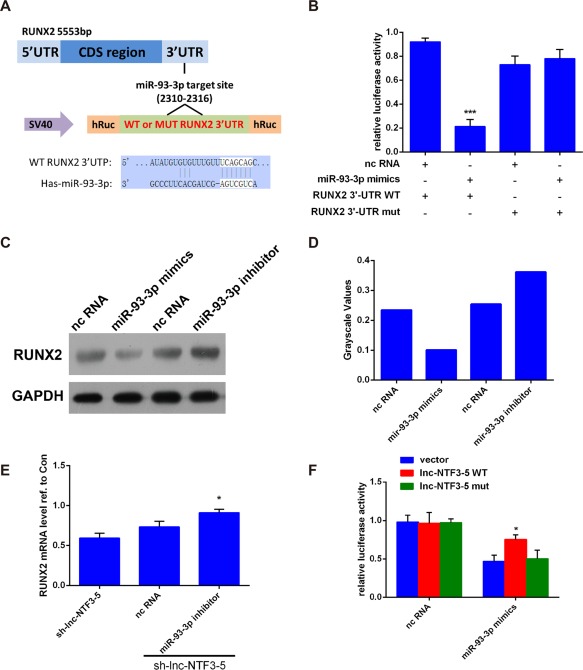
MiR‐93‐3p downregulated the expression of RUNX2. (A) Prediction of binding sites of miR‐93‐3p in RUNX2. (B) The 293T cells were cotransfected with miR‐93‐3p mimics or nc RNA and the luciferase constructs carrying RUNX2 WT 3′ UTR or Mut 3′ UTR. Luciferase activity was tested 48 h after transfection. (C) Western blot analysis of RUNX2 protein expression in MSMSCs transfected with miR‐93‐3p mimics or inhibitor. (D) Results of western blotting were analyzed with Gel Image system software (Tanon, Shanghai, China) and data were presented as ratio of target protein to GAPDH in the form of grayscale value. (E) The miR‐93‐3p inhibitor rescued the sh‐lnc‐NTF3–5‐mediated downregulation of RUNX2 expression. (F) The luciferase of RUNX2, which inhibited by miR‐93‐3p mimics, was significantly rescued after cotransfection of lnc‐NTF3–5 WT, compared with vector or lnc‐NTF3–5 Mut (*P < .05)

To further explore the mechanism of lnc‐NTF3–5 promote osteogenic differentiation, Target Scan and miRDB were used to analysis the binding sites of miR‐93‐3p on the osteogenic master gene *RUNX2*. Results showed that 3′ UTR of the human RUNX2 mRNA has a putative binding site for miR‐93‐3p within the 2310–2316 nt (Figure 5A). To identify the miR‐93‐3p target region in RUNX2 mRNA, a luciferase assay was employed. RUNX2 3′ UTR luciferase reporter containing miR‐93‐3p binding site was constructed and transfected into 293T cells. Results demonstrated that miR‐93‐3p decreased RUNX2‐WT 3′ UTR luciferase reporter activity in 293T cells, whereas the mutation of RUNX2 3′ UTR binding site abolished the repression induced by miR‐93‐3p (Figures 5B), indicating that the predicted sequence is the functional miR‐93‐3p binding site. The protein expression levels of RUNX2 in response to miR‐93‐3p were analyzed. As shown in Figure 5C and D, RUNX2 protein levels were significantly decreased by the treatment with the miR‐93‐3p mimics and increased by the treatment with the miR‐93‐3p inhibitor. Taken together, these results indicated that miR‐93‐3p inhibits RUNX2 expression through direct binding to the target site in the RUNX2–3′ UTR.

### 3.6 Lnc‐NTF3–5 regulated RUNX2 expression through sponging miR‐93‐3p

Lnc‐NTF3–5 may function as miR‐93‐3p sponges to regulate the expression of RUNX2 to affect osteogenic differentiation of MSMSCs. To further explore how lnc‐NTF3–5 exert its function, we next transferred miR‐93‐3p inhibitor into lnc‐NTF3–5 downregulated MSMSCs. In comparison with the sh‐lnc‐NTF3–5 group, co‐transfection of miR‐93‐3p inhibitor with sh‐lnc‐NTF3–5 significantly rescued RUNX2 mRNA expression (Figures 5E). Besides, the RUNX2 luciferase reporter and lnc‐NTF3–5 wild‐type, lnc‐NTF3–5 mutant or vector were co‐transferred into 293T cells contain miR‐93‐3p mimics. Results showed that miR‐93‐3p mimics could suppress the luciferase activity of RUNX2, whether 293T cells were transfected by lnc‐NTF3–5 WT or by lnc‐NTF3–5 Mut (Figures 5F). Nevertheless, in comparison with the vector and lnc‐NTF3–5 mut groups, transfection of lnc‐NTF3–5 WT could significantly restore the activity of RUNX2 luciferase (Figures 5F).

### 3.7 Lnc‐NTF3–5 increased in vivo ectopic bone formation in human MSMSCs

**Figure 6:**
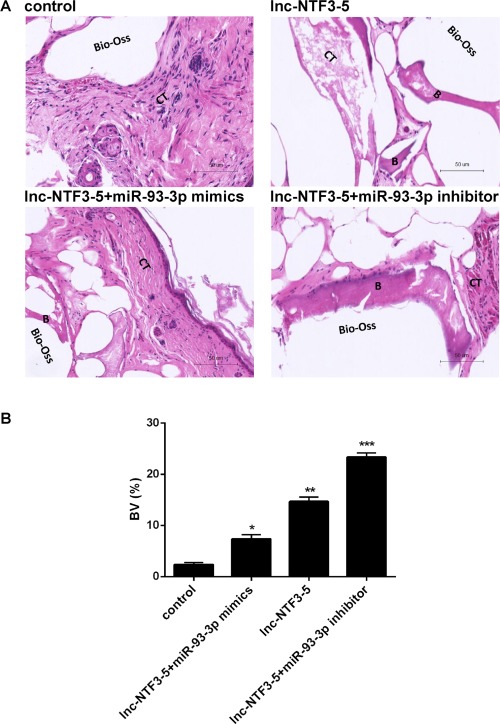
Lnc‐NTF3–5 increased *in vivo* ectopic bone formation in MSMSCs. (A) Representative image from each sample. B, bone; CT, connective tissue. (B) Bone volume (BV) was quantified using Image Pro 5.0 system (Tanon, Shanghai, China). Bone volume = new bone area/total section size (*P< .05, **P< .01, ***P< .001)

The results described above demonstrated that lnc‐NTF3–5 may function as miR‐93‐3p sponges to regulate osteogenic differentiation of MSMSCs in vitro, we next explored whether the regulation of the lnc‐NTF3–5 and miR‐93‐3p expression levels in MSMSCs also exerted an effect on ectopic bone formation in vivo. The regenerated new bone was observed after 8 wk transplantation, when there were nodules in graft. The Lnc‐NTF3–5 and miR‐93‐3p inhibitor co‐transfection group showed the strongest bone formation than the other groups (Figure 6A, B). Whereas co‐transfection of lnc‐NTF3–5 and miR‐93‐3p mimics impaired bone formation in contrast to the lnc‐NTF3–5 transfection group (Figure 6A, B). These results indicated that lnc‐NTF3–5 could promote ectopic bone formation while miR‐93‐3p suppresses the effect.

## 4 DISCUSSION

LncRNAs are generally defined as transcripts of longer than 200 nucleotides without evident of coding proteins and can regulate diverse cellular processes.^21^ They are expressed at significantly lower levels but more cell type specific than protein‐coding RNAs.^22^ Our recent study found that lncRNAs can affect the osteogenic differentiation of MSMSCs, the top 5 lncRNAs were validated by qRT‐PCR.^19^ LncRNA‐MODR plays a positive role in osteogenic differentiation of MSMSCs.^19^ Similarly, researchers found that lncRNA‐MEG can promote osteogenic differentiation as well.^17^ This osteogenic promotion effect was demonstrated in human adipose‐derived mesenchymal stem cells (ADMSCs) recently.^23^. The current study found that lnc‐NTF3–5 was able to enhance RUNX2, OSX, and ALP expression and further promoted the osteogenic differentiation of MSMSCs, which suggested that lnc‐NTF3–5 may have a similar function as lncRNA‐MODR and lncRNA‐MEG. However, a recent study demonstrated that lncRNA‐MEG3 inhibited osteogenic differentiation of bone marrow mesenchymal stem cells (BM‐MSCs).^24^ Meanwhile, MEG3 expression was decreased significantly in the first 6 d of differentiation, but slowly raised in the following days.^24^ Similarly, knockdown of HoxA‐AS3 or MIR31HG resulted in an enhanced osteogenic differentiation in MSCs^18^ and ADMSCs.^25^ Taken together, these studies suggested that lncRNAs play an essential role in osteogenic differentiation of stem cells whereas their function are cell specific and pathological‐dependent, which needs to be clarified.

To further confirm the function of lnc‐NTF3–5 in osteogenic differentiation of MSMSCs, loss‐ and gain‐of‐function strategy was employed. Lnc‐NTF3–5 overexpression and knockdown MSMSCs were then constructed and subjected to osteogenic differentiation. As is well known, RUNX2 is the core transcription factor in controlling osteogenic differentiation.^26^ RUNX2 can directly stimulate transcription of osteoblast‐related gene OSX and ALP.^27^, ^28^ They were significantly upregulated in the lnc‐NTF3–5‐overexpression group. On the contrary, these markers were significantly downregulated in the lnc‐NTF3–5‐knockdown group. In addition, lnc‐NTF3–5‐overexpression enhanced new bone formation when MSMSCs were transplanted with Bio‐Oss scaffold. These results indicated that lnc‐NTF3–5 promoted the osteogenesis of MSMSCs both in vitro and in vivo.

There is now evidence that lncRNAs regulate gene expression and function at both transcriptional and post‐transcriptional levels.^29^ Four different mechanisms have been reported in lncRNAs and miRNAs interaction: miRNAs can bind to lncRNAs and reduce its stability; lncRNAs can act as sponges of miRNAs; lncRNAs can compete with miRNAs for binding to target mRNAs; and lncRNAs can generate miRNAs.^30^ The interaction between lncRNAs and miRNAs represents a new aspect of physiological and pathological cellular process, which draws more and more attentions.^30^ For instance, lncRNA H19 was reported to modulate a panel of Notch‐associated miRNAs during BMP9‐induced osteogenic differentiation of MSC cell lines.^31^ Another lncRNA, MALAT1, could directly interact with miR‐204 and promoted osteogenic differentiation of human aortic valve interstitial cells.^32^ Bioinformatics analysis results indicate that miR‐93‐3p can bind to lnc‐NTF3–5. Thus, we further explored the relation between miR‐93‐3p and lnc‐NTF3–5. Our results demonstrated a strong negative correlation of miR‐93‐3p expression with lnc‐NTF3–5. On one hand, this study showed that overexpression of lnc‐NTF3–5 decreased miR‐93‐3p while lnc‐NTF3–5 inhibition elevated miR‐93‐3p expression. On the other hand, miR‐93‐3p mimics reduced lnc‐NTF3–5 expression level whereas miR‐93‐3p inhibitor strengthened lnc‐NTF3–5 expression level. These results suggested that lnc‐NTF3–5 may have a similar function as lncRNA‐MODR.^19^

Many studies suggested that miRNAs can directly target the osteogenic master regulator RUNX2.^33^ To elucidate the role of lnc‐NTF3–5 in regulating osteogenic differentiation, bioinformatics analysis was performed to predict whether miR‐93‐3p can bind to the key transcription factor for osteogenesis RUNX2. Results found that miR‐93‐3p can directly bind to 2310–2316 nt of RUNX2 3′ UTR, which suggested that miR‐93‐3p may regulate RUNX2 expression. Previous studies revealed that a plenty of miRNAs were involved in suppressing osteogenesis by targeting RUNX2, such as miR‐103a,^34^ miR‐133a,^35^ miR‐135a,^33^ miR‐204/211,^36^ and miR‐217.^37^ miR‐93‐3p was reported to be upregulated in HIV‐associated neurocognitive‐disordered patients,^38^ while downregulated in radioresistant nasopharyngeal carcinoma cells.^39^ Meanwhile, miR‐93‐3p was suggested to be a potential biomarker of acute kidney injury (AKI) in intensive care units (ICU), and cardiac surgery (CS) patients since it was significantly downregulated during the days prior to AKI diagnosis.^40^. However, the function of miR‐93‐3p in osteogenic differentiation is still unknown. This study not only first demonstrated that miR‐93‐3p acts as a negative regulator of osteogenic differentiation but also extended the RUNX2‐targeting miRNAs family by showing that miR‐93‐3p strongly inhibit RUNX2 expression at both mRNA and protein levels.

Since lnc‐NTF3–5 functions as miR‐93‐3p sponges, we next explored whether the regulation of the lnc‐NTF3–5 and miR‐93‐3p in MSMSCs also exerts an effect on bone formation in vivo. Results suggested that lnc‐NTF3–5 overexpression led to significant new bone formation in mice. Moreover, co‐transfection of miR‐93‐3p inhibitor led to an even enhanced bone formation in mice. These data suggest that lnc‐NTF3–5 promoted bone formation through competitively binding to miR‐93‐3p in vivo.

In conclusion, this study first characterized the osteogenic promoting function of lnc‐NTF3–5 in MSMSCs and demonstrated that lncRNA‐microRNA crosstalk may be the underlying mechanism. Lnc‐NTF3–5 competitively binding to miR‐93‐3p promotes the expression of RUNX2. This study indicated lnc‐NTF3–5 might be a novel biomarker or therapeutic target for promoting bone regeneration in the posterior maxilla.

## CONFLICT OF INTEREST

The authors declare that they have no conflicts of interest with the contents of this article.
